# Effects of the 2-Repeat Allele of the DRD4 Gene on Neural Networks Associated With the Prefrontal Cortex in Children With ADHD

**DOI:** 10.3389/fnhum.2018.00279

**Published:** 2018-07-12

**Authors:** Andan Qian, Jiejie Tao, Xin Wang, Huiru Liu, Lingxiao Ji, Chuang Yang, Qiong Ye, Chengchun Chen, Jiance Li, Jingliang Cheng, Meihao Wang, Ke Zhao

**Affiliations:** ^1^Department of Radiology, First Affiliated Hospital of Wenzhou Medical University, Wenzhou, China; ^2^Department of Radiology, Yancheng First Peoples’ Hospital, Yancheng, China; ^3^Department of Mental Health, First Affiliated Hospital of Wenzhou Medical University, Wenzhou, China; ^4^Department of Anatomy, Wenzhou Medical University, Wenzhou, China; ^5^Department of Radiology, First Affiliated Hospital of Zhengzhou University, Zhengzhou, China; ^6^School of Mental Health, Wenzhou Medical University, Wenzhou, China

**Keywords:** attention-deficit hyperactivity disorder, functional magnetic resonance imaging, DRD4, 2-repeat allele, group independent component analysis

## Abstract

**Objective**: Genetic variation, especially polymorphism of the dopamine D4 receptor gene (DRD4), has been linked to deficits in self-regulation and executive functions and to attention deficit hyperactivity disorder (ADHD), and is related to the structural and functional integrity of the default mode network (DMN), the executive control network (ECN) and the sensorimotor network (SMN). The aim of this study was to explore the effects of the 2-repeat allele of the DRD4 gene on brain network connectivity and behaviors in children with ADHD.

**Methods**: Using independent component analysis (ICA) and dimension analyses, we examined resting-state functional magnetic resonance imaging (fMRI) data obtained from 52 Asian medicine-naive children with ADHD (33 2-repeat absent and 19 2-repeat present).

**Results**: We found that individuals with 2-repeat absent demonstrated increased within-network connectivity in the right precuneus of the DMN, the right middle frontal gyrus (MFG) of the SMN compared with individuals with 2-repeat present. Within the ECN, 2-repeat absent showed decreased within-network connectivity in the left inferior frontal gyrus (IFG) and the left anterior cingulate cortex. A deeper study found that connectivity strength of the left IFG was directly proportional to the Stroop reaction time in 2-repeat absent group, and as well as the right MFG in 2-repeat present group.

**Conclusion**: Polymorphisms of the DRD4 gene, specifically 2-repeat allele, had effects on the ECN, the SMN and the DMN, especially in the prefrontal cortex (PFC) circles. ADHD children with DRD4 2-repeat allele have aberrant resting-state within-network connectivity patterns in the left IFG and the right MFG related to dysfunction in inattention symptom. This study provided novel insights into the neural mechanisms underlying the effects of DRD4 2-repeat allele on ADHD.

## Introduction

Attention deficit hyperactivity disorder (ADHD) is a multi-factorial, polygenetic disorder, characterized by inattention, impulsivity and hyperactivity, with a worldwide prevalence of 5.3% (Polanczyk et al., 2007). ADHD is associated with neuropsychological deficits, academic difficulties and behavior problems that lead to social and mental issues (Able et al., 2007; Lingam et al., 2012). It was reported that the genetic heritability of ADHD is as high as 75%, and environmental factors are estimated to account for 25% of the development of ADHD (Faraone et al., 2005). The leading candidate gene was dopamine D4 receptor gene (DRD4), which is the strongest and most consistently replicated gene related to ADHD (Banaschewski et al., 2010). A frequently studied polymorphism of the DRD4 gene is a 48 base-pair (bp) variable number of tandem repeats (VNTR) located on exon III. The number of repeats varies widely, from 2 to 11, but alleles with 2, 4 and 7 repeats are the most common (Ding et al., 2002). Molecular genetic studies showed that the DRD4 gene regulates the efficiency of the central dopaminergic pathways. In comparison with 4-repeat alleles, 7-repeat alleles exhibited a suboptimal response to dopamine, whereas 2-repeat alleles exhibited an intermediate profile (Asghari et al., 1995; Oak et al., 2000).

From the perspective of epidemiology, converging research findings indicated that carriers of alleles with long-repeats (ranging from 4 to 7) were at an increased risk of developing ADHD in Western and Asian populations (Qian et al., 2004, 2007; Faraone et al., 2005; Gizer et al., 2009). A small study with a Chinese population showed a high frequency of 2-repeat allele in ADHD (Leung et al., 2005). Furthermore, Asians, including Chinese, are known to rarely exhibit the 7-repeat allele, with 2-repeat allele being more prevalent (Kitayama et al., 2014; Park et al., 2014). It was proposed that 2-repeat allele acted as a substitute of 7-repeat allele in Asian populations (Reist et al., 2007). However, neuropsychological studies investigating the connection between ADHD and behaviors associated with a 2-repeat allele of DRD4 produced heterogeneous results. It was reported that subjects with short repeats (2–5) performed significantly worse on attention tests than those with long repeats (6–8; Manor et al., 2002). Nevertheless, in consideration of gene × culture interaction, individuals with 2/7-repeat allele heterozygous genotype might show behaviors that were considered culturally sanctioned social orientations (Kitayama et al., 2014). The DRD4 2-repeat was significantly associated with a more stable perception (Kang et al., 2008; Schmack et al., 2013).

Genetic variation is more intuitive to express the individual differences in brain activity, even in the absence of differences in behaviors. Thus, neuroimaging could act as an intermediate phenotype to investigate the effect of genes on behavior, and several studies have used this tool to show that individuals with different genotypes display differential patterns of brain function and structure (Durston et al., 2005; Klein et al., 2017). For example, using the task functional magnetic resonance imaging (fMRI) technique, Gilsbach et al. (2012) illustrated that DRD4 polymorphism affected the neural networks associated with executive function performances in healthy subjects. In some studies involving ADHD patients, carriers of DRD4 7-repeat allele showed thinner parietal cortex (Shaw et al., 2007). Recently, only two resting-state fMRI studies implicated that DRD4 2-repeat may also alter the local brain functional activity of the parietal or prefrontal regions in children with ADHD and have found that these abnormalities would be related to dysfunction in a sustained and divided attention test (Kim et al., 2018; Qian et al., 2018). Thus, DRD4 polymorphisms may have an effect on brain structure and activity, even though existing evidences did not support firm conclusions about the pathways from gene to disease, DRD4 polymorphisms may have an effect on brain structure and activity.

Furthermore, our investigation focused on in brain regions where genes were preferentially expressed to verify their effects. DRD4 gene expression levels were relatively increased in several brain areas related to planning and rewarding, including the prefrontal cortex (PFC), cingulate gyrus and striatum. These brain regions also played an important role in regulating executive functions (Seeman et al., 1993; Mulcrone and Kerwin, 1997; Gehricke et al., 2015). For example, DRD4 polymorphisms presented different blood oxygen level dependency (BOLD) responses in the PFC and cingulate gyrus during tasks of executive function (Gilsbach et al., 2012). So, we investigated brain regions with a high dopaminergic receptor density and that were involved in cognitive-related functional networks, including the primary perception network (the sensorimotor network (SMN)) and advanced cognition network (the default mode network (DMN), the executive control network (ECN); Posner et al., 2014). From the above three factors, we hypothesized that the DRD4 2-repeat allele could modulate brain networks of ADHD patients which may underlie the association between this gene and hyperactivity-impulsivity behaviors or the executive tasks.

In the present study, we applied an independent component analysis (ICA), a hypothesis-free analysis of whole-brain fMRI data. In contrast to traditional region-of-interest (ROI) seed-based correlation method, ICA can capture signals from linear mixtures of these signals and then apply higher-order signal statistics to determine a set of “components” that are maximally independent of each other (Mantini et al., 2007). This type of analysis thus reduces the loss of some features due to data correlation analysis using a pre-assumption model (or a model established from healthy population) to fit real data in seed-based correlation approaches. ICA is also able to extract noise from a desired dataset and is thus a powerful method for detecting interactions within network regions (Zuo et al., 2010; Hoekzema et al., 2014).

In previous studies, the role of 2-repeat allele was always tied to 7-repeat allele for several neuroimaging and neuropsychology studies (Wang et al., 2004; Henríquez-Henríquez et al., 2015); however, the independent effect of 2-repeat allele was neglected. On the basis of our research on the local brain activity (Qian et al., 2018), a whole brain analysis of ADHD may contribute to a more comprehensive examination of the underlying neural mechanisms. To the best of our knowledge, little is known about the effect of DRD4 2-repeat allele on the brain networks of ADHD. Thus, we selected 2-repeat allele as an intermediate profile for our study, involving a group of only medicine-naïve patients with ADHD, and applied ICA, a data-driven approach to study whole-brain fMRI data.

## Materials and Methods

### Participants

We recruited 72 Chinese Han children with ADHD from the department of psychiatry from the First Affiliated Hospital of Wenzhou Medical University in China. The recruited children were between 7 years and 14 years old and were diagnosed by three experienced psychiatrists in accordance with the criteria of Diagnostic and Statistical Manual of Mental Disorders, 4th Revision (DSM-IV).

Thirteen subjects were excluded from further analysis. The exclusion criteria are as follows: (1) a history of organic brain diseases and head trauma with disturbance of consciousness; (2) a history of psychiatric disorders according to the Schedule for Affective Disorders and Schizophrenia for School-Age Children-Present and Lifetime Version, (K-SADS-PL); (3) an intelligence quotient (IQ) below 75 according to the Chinese revised Wechsler Intelligence Scale; (4) any previous course of psychotropic medications treatment; and (5) substance dependency or substance abuse (including heroin, nicotine, or alcohol addiction).

All procedures performed in studies involving human participants were in accordance with the ethical standards of the clinical ethics committee of the First Affiliated Hospital of Wenzhou Medical University and with the 1964 Helsinki declaration and its later amendments or comparable ethical standards. All participants provided written informed consent after receiving a full explanation of the test procedure. All subjects participated voluntarily and were informed of the purposes, methods, and potential risks of the study, and an informed consent form was signed by parents/guardians. Simultaneously, the parents and children were instructed to complete the “Conners Parent Symptom Questionnaire (PSQ),” “Stroop Color and Word Test (SCWT)” and “Wisconsin Card Sorting Test (WCST).”

### Genotyping

Blood samples were obtained by venipuncture at the bend of the elbow, and genomic DNA extracted from the samples was analyzed using TIANGEN (NO. DP304). Then, we genotyped the DRD4 Exon-3 48 bp VNTR of each subject using polymerase chain reaction (PCR) and short tandem repeat (STR) methods. The primers used in this protocol are as follows: forward: 5’-GCG ACT ACG TGG TCT ACT CG-3’, reverse: 5’-AGG ACC CTC ATG GCC TTG-3’. PCR reactions were prepared in 25 μL volumes, each containing 12.5 μL KAPA2G Fast Multiplex Mix, 1 μL Forward primer (10 μm), 1 μL Reverse primer (10 μm), 1 μL Template DNA and 9.5 μL ddH2O. The samples were heated at 95°C for 10 min and then cycled 40 times at 94°C for 30 s, 55°C for 30 s and 72°C for 1 min, followed by 72°C for 3 min with a final extension at 4°C.

The GS500LIZ standard sample was mixed with the PCR products and subjected to HIDI for electrophoresis to obtain the atlas of STR. All participants were genotyped for the DRD4 48-bp repeat VNTR polymorphism.

### Image Acquisition

Whole-brain imaging was performed on a GE Signa HDx 3.0 Tesla MR scanner (USA). For exclusion of intracranial structural lesions, T2 weighted images were collected (repetition time (TR)/echo time (TE) = 2000/102 ms; field of view (FOV) = 240 mm × 240 mm; slice thickness = 5.0 mm; no gap; 25 transversal slices) prior to the collection of the resting-state fMRI data. Resting-state fMRI data were obtained using an Echo-Planar Imaging sequence (EPI) with the following imaging parameters: TR/TE = 2000/30 ms; FOV = 192 mm × 192 mm; matrix = 64 × 64; flip angle (FA) = 90°; slice thickness = 4 mm; slice gap = 0.2 mm; 31 transversal slices; and 240 volumes. During the fMRI scans, all subjects were instructed to keep their eyes closed and to wear sponge earplugs, to stay as still as possible, to think of nothing in particular, and to remain awake. To ensure the children’s safety, they were accompanied by a guardian for the full scan. The experimenters who collected the fMRI data were blinded to the results of genotyping at the time of data collection.

### fMRI Data Preprocessing

There were no artifacts or structural abnormalities in the structural MRI images, as determined by visual inspection. The resting-state fMRI data preprocessing steps were conducted using Data Processing Assistant for Resting-State fMRI (DPARSF; Chao-Gan and Yu-Feng, 2010) on MATLAB 7.12.0 (R2013a). The first 10 time points of each functional time series were discarded to remove non-steady-state effects. The remaining 230 time points were corrected for the acquisition time delay between different slices. Then, a voxel-specific head motion calculation was included to the head motion correction, which performed frame-wise displacement measurements (FD) of head motion as introduced by Power et al. (2013). So, a threshold for head motion during this study was established at ± 3.0 mm and/or ± 3.0° shift during the experiment together with the requirement that no volume in a subject was allowed to have an FD value over 0.5. To these thresholds of motion, we excluded seven subjects in process of head motion realignment. Subsequently, the images were spatially normalized to a standard template (Montreal Neurological Institute, Montreal, QC, Canada) and resampled to 3 mm × 3 mm × 3 mm voxels. The normalized fMRI data were smoothed with 4-mm full width at half-maximum (FWHM) Gaussian kernel to accommodate inter-subject anatomical variability.

### Group Independent Component Analysis (GICA)

**Figure 1:**
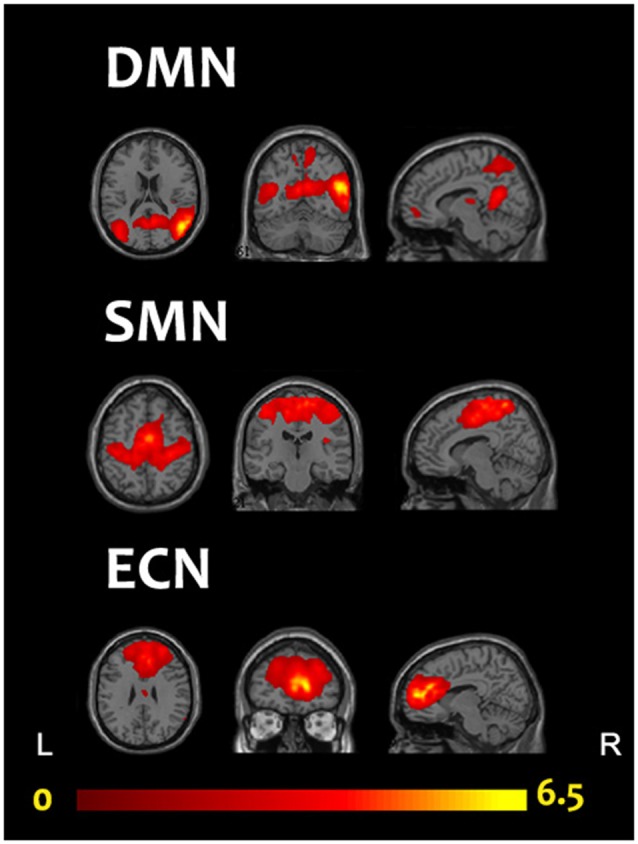
Three components of interest from group ICA represent networks of interest (red). Data are displayed on the three most informative orthogonal slices for each network. Color bar represents Z-values ranging from 0 to 6.5. ICA, independent component analysis; DMN, default mode network; ECN, executive control network; SMN, sensorimotor network; L, left; R, right.

We adopted the group ICA (GICA) of the fMRI toolbox, MICA beta1.22^1^ (Zhang et al., 2010) to obtain more stable results. Zhang et al. (2010) found that for multistage principal component analysis (PCA) reduction, which is adopted and implemented in GIFT and MELODIC, different subject concatenation orders (SCOs) produce unstable GICA results. Therefore, to obtain more stable and accurate results, Zhang et al. (2010) proposed an improved algorithm, the subject order-independent group ICA (SOI-GICA), which was adopted in MICA, each time with randomized initial values and a different subject order. Then, the multiple results were integrated to form the final output of MICA (Zhang et al., 2010). The fMRI toolbox performed the analysis in the following three main stages: (1) data reduction (PCA); (2) application of the ICA algorithm; and (3) back-reconstruction for each individual subject. In our study, we adopted the SOI-GICA and performed GICA 100 times to obtain 30 independent components for each subject according to previous studies (Maneshi et al., 2014; Liang et al., 2015; Reineberg et al., 2015). Visual inspection of discarded components suggested that they were likely related to eye movement, head motion, or heart-beat induced cerebral pulsation. Three meaningful components were identified by visual inspection, including the DMN, the ECN and the SMN, which were found to be highly consistent with previous studies in terms of network nodes (see Figure 1; Damoiseaux et al., 2006; Smith et al., 2009). Subject-specific spatial maps were acquired and z-score transformed before group comparisons.

### Statistical Analyses

The brain resting-state fMRI maps of the two groups were analyzed using REST software (version 1.8). First, each ICA component was entered into a one-sample *t*-test to generate a sample-specific spatial mask for each component; the sample here referred to both 2-repeat absent and 2-repeat present groups. Note that they were two-tailed *t*-tests but we only retained positive-sided results (i.e., t > t-threshold) as what lots of previous ICA studies have done (Roosendaal et al., 2010; Liang et al., 2015). A sample-specific spatial mask was served as a mask for following analyses (Luo et al., 2012). Subsequently, two-sample *t*-tests were conducted within the predefined mask to examine the differences within each network between the two groups with gender, age, IQ and head motion as nuisance covariates. Thresholds were set at *p* < 0.05 with a minimum cluster size, which corresponded with AlphaSim multiple comparison correction *p* < 0.001 (using the impact of smoothing and cluster-defining threshold (CDT) with the parameters including: 5000 simulations, full width at half maximum = 4 mm, cluster connections radius = 5 mm; Eklund et al., 2016).

### ADHD Dimension Analysis

The Impulsivity-Hyperactivity (IH) measured hyperactivity and impulsivity symptoms and were obtained from Conners PSQ Chinese revised version (Goyette et al., 1978). SCWT, especially in their reaction times of response (RT), was one of the most consistent manifestations of the ADHD (Buzy et al., 2009) and served as a reliable predictor of diagnostic severity (Kuntsi et al., 2010). The WCST was a standard test used to assess various aspects of cognitive functions and executive functions.

We performed correlation analyses with performance on the SCWT task and on the WCST task, an index of selective attention and executive function, to assess whether activity in brain regions that showed a different pattern of recruitment across the entire sample was associated with attention or executive performance. Subsequently, an explorative partial correlation analysis was performed to test whether or not correlations go in different directions in the two genotype groups. Pearson’s correlation or Spearman’s correlation between the ROIs of ICA results and the behavior items were analyzed using SPSS software (version 22.0), with a *p* value < 0.05 considered statistically significant. Bonferroni corrections were then used for multiple comparisons.

## Results

### Demographics of the Sample

The participants were divided into two groups according to genotype (2-repeat absent: *n* = 33; 29 with 4R/4R genotype, four with a 5R/4R genotype; 2-repeat present: *n* = 19; 15 with 2R/4R genotype, one with a 2R/5R genotype, one with a 2R/3R genotype, one with a 2R/2R genotype). The genotypes were in Hardy-Weinberg equilibrium. Table 1 summarizes the characteristics of the samples. The groups did not differ significantly with regard to age (*p* = 0.060), IQ (*p* = 0.689) or sex distribution (*p* = 0.782).

**Table 1:** Demographic and clinical characteristics of dopamine D4 receptor gene (DRD4) groups.

Characteristic	2-repeat absent *N* = 33	2-repeat present *N* = 19	Test statistic	*P* value
Age (Mean ± SD years)	9.91 ± 1.94	8.84 ± 1.89	*T* = 1.925	0.060
Gender (female/male)	3/30	3/16	*χ*^2^ = 0.077	0.782
IQ (Mean ± SD)	117.09 ± 16.74	115.26 ± 13.79	*T* = 0.403	0.689
Handedness (right/left)	33/0	19/0		
ADHD subtype				
(inattentive/hyperactivity-impulsivity/combined)	17/4/12	6/4/9	*χ*^2^ = 2.070	0.355
Conners parent symptom questionnaire				
Conners index of hyperactivity	1.48 ± 0.52	1.61 ± 0.62	*T* = −0.785	0.436
Impulsivity-hyperactivity (IH)	1.53 ± 0.61	1.70 ± 0.70	*T* = −0.902	0.371
Anxiety	0.49 ± 0.07	0.50 ± 0.06	*T* = −0.862	0.393
Physical and psychological problems	0.37 ± 0.29	0.41 ± 0.37	*T* = 1.348	0.184
Stroop color and word test (SCWT)				
Stroop ignored numbers	43.18 ± 21.76	46.79 ± 21.42	*T* = −0.579	0.565
Stroop reaction time (RT)(s)	2.73 ± 1.06	2.83 ± 1.09	*T* = −0.335	0.739
Wisconsin card sorting test (WCST)				
Perseverative errors (WCST-P)	15.76 ± 9.31	15.68 ± 9.68	*T* = 0.027	0.979
Nonperseverative errors (WCST-NP)	14.18 ± 6.50	12.58 ± 6.25	*T* = 0.868	0.390

*SD, standard deviation*.

### ICA Connectivity Differences in Brain Networks Between the DRD4 Genotypes

**Figure 2:**
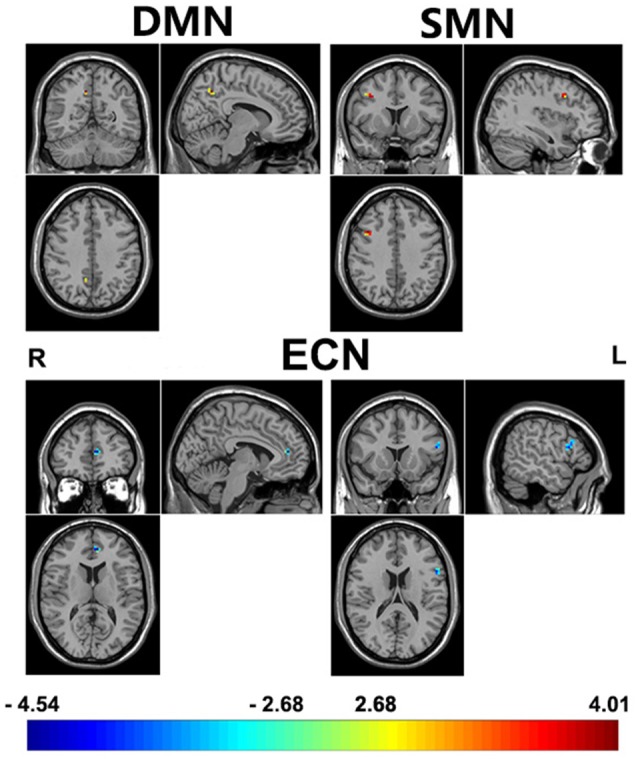
A comparison of ICA results between 2-repeat absent group and 2-repeat present group. The threshold was set at a AlphaSim corrected *p* < 0.001. Color bar represents T-values ranging from −4.54 to 4.01. These regions are the right precuneus (9, −57, 42, MNI coordinates) in the DMN, the right middle frontal gyrus (MFG; 39, 12, 39) in the SMN, the left inferior frontal gyrus (IFG; −54, 15, 18) and the left anterior cingulate cortex (−6, 45, 12) in the ECN. DMN, default mode network; ECN, executive control network; SMN, sensorimotor network; L, left; R, right.

**Figure 3:**
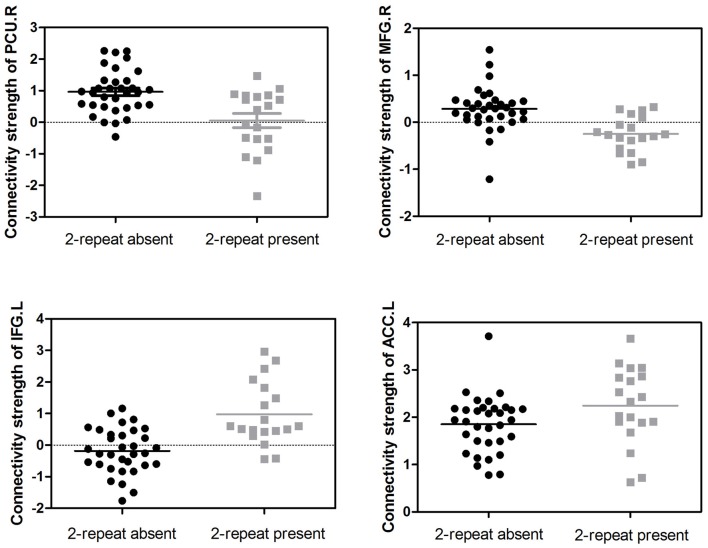
Distributions of the connectivity strength (z values) are shown for each cluster in the DMN, the SMN and the ECN between the two groups. PCU, precuneus; MFG, middle frontal gyrus; IFG, inferior frontal gyrus; ACC, anterior cingulate cortex; L, left; R, right.

Adjusting for age, sex, IQ and head motion as covariates emphasized the differences between the ADHD 2-repeat allele absent and the 2-repeat allele present groups. Compared with the 2-repeat present group, the 2-repeat absent group showed increased within-network connectivity in the right precuneus (PCU) of the DMN (peak MNI coordinates: *x* = 9, *y* = −57, *z* = 42). Similarly, the 2-repeat absent group showed significantly increased within-network connectivity in the right middle frontal gyrus (MFG) of the SMN (*x* = 39, *y* = 12, *z* = 39). Within the ECN, the 2-repeat absent group showed decreased within-network connectivity in the left inferior frontal gyrus (IFG; *x* = −54, *y* = 15, *z* = 18) and the left anterior cingulate cortex (ACC; *x* = −6, *y* = 45, *z* = 12). There were significant within-network connectivity differences (*p* < 0.001, AlphaSim correction) in the DMN, the SMN and the ECN between the two groups (see Table 2 and Figures 2, 3).

**Table 2:** Brain areas with independent component analysis (ICA) connectivity differences between 2-repeat absent and 2-repeat present groups.

Network	Non *2* > 2	2 > Non 2	*P*-value	*T*-value	Cluster size	Brain regions
DMN	9, −57, 42		0.0002	4.013	5	Precuneus R
ECN		−54, 15, 18	0.0001	−4.4702	18	Inferior Frontal Gyrus L
		−6, 45, 12	0.0001	−4.6513	8	Anterior Cingulate Cortex L
SMN	39, 12, 39		0.0002	4.0121	11	Middle Frontal Gyrus R

*L, left; R, right; Coordinates (x, y and z) are given in mm of MNI template space*.

### Dimensional Analyses

**Figure 4:**
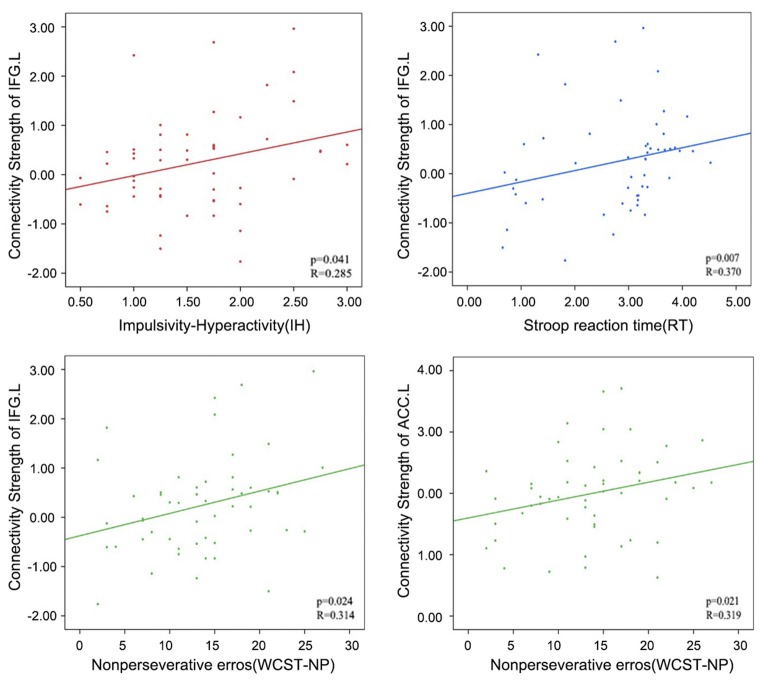
Correlations between the connectivity strength (z values) of left IFG or left ACC in the ECN and the behavioral characteristics in attention deficit hyperactivity disorder (ADHD) patients. PCU, precuneus; MFG, middle frontal gyrus; IFG, inferior frontal gyrus; ACC, anterior cingulate cortex; L, left; R, right.

In addition, relatively little is known regarding how altered within-network connectivity measured during resting state is related to behaviors. In the ECN, the left IFG showed the underlying trends of positive correlations between connectivity strength (z values) and the IH (Pearson’s correlation, *p* = 0.041), RT (Spearman’s correlation, *p* = 0.007) and WCST-NP (Spearman’s correlation, *p* = 0.024); as well as the left ACC between connectivity strength and the WCST-NP (*p* = 0.021). However, no significant results remained following Bonferroni corrections (*p* < 0.05/12 = 0.004; uncorrected results given in Table 3 and Figure 4).

**Table 3:** Effects for positive correlation between behavioral characteristics and connectivity strength in the executive control network.

Brain regions	Behavioral characteristics	*P*-value	*R*-value^a^
Inferior Frontal Gyrus L	Impulsivity-hyperactivity (IH)	0.041^b^	0.285
Inferior Frontal Gyrus L	Stroop reaction time (RT)	0.007^c^	0.370
Inferior Frontal Gyrus L	Nonperseverative errors (WCST-NP)	0.024^c^	0.314
Anterior Cingulate Cortex L	Nonperseverative errors (WCST-NP)	0.021^c^	0.319

*^a^R-Values reflect the correlation coefficient between the peak voxel connectivity value and behavioral characteristics. ^b^Pearson’s correlation. ^c^Spearman’s correlation*.

**Figure 5:**
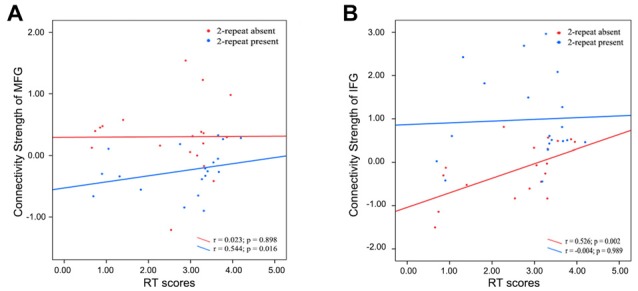
Partial correlations between the connectivity strength (z values) of specific regions and the RT scores in 2-repeat absent group and 2-repeat present group. RT, Stroop reaction time. **(A)** A correlation between the connectivity strength of the right MFG in the SMN and the RT scores. MFG, middle frontal gyrus; SMN, sensorimotor network. **(B)** A correlation between the connectivity strength of left IFG in the ECN and the RT scores. IFG, inferior frontal gyrus; ECN, executive control network.

Spearman correlation analysis was also performed between connectivity strength (z values) of the left IFG or the right MFG in both two genotype groups and above three scores. In the 2-repeat absent group, connectivity strength of the IFG (Spearman’s correlation, *r* = 0.526, *p* = 0.002, *p* < 0.05/12 = 0.004, Bonferroni correction) were directly proportional to the RT scores. No correlation between the RT scores and connectivity strength of the IFG in the 2-repeat present group (Spearman’s correlation, *r* = −0.004, *p* = 0.989; see Figure 5). The left ACC failed in partial correlation analyses. In the 2-repeat present group, connectivity strength of the right MFG in the SMN were prone to have correlation to the RT scores (Spearman’s correlation, *r* = 0.544, *p* = 0.016). But no significant result remained following Bonferroni correction (*p* < 0.05/12 = 0.004). No correlation between the RT scores and connectivity strength of the right MFG in the 2-repeat absent group was found (Spearman’s correlation, *r* = 0.023, *p* = 0.898; see Figure 5). There was no statistical difference in the IH, in the RT and in the WCST-NP of the two genotype groups (see Table 1).

## Discussion

The ECN covers the superior and middle prefrontal cortices as well as several medial-frontal areas, including the anterior cingulate and paracingulate sulci, which strongly correspond to several cognition paradigms and to action-inhibition, emotion, and perception-somesthesis-pain (Beckmann et al., 2005; Damoiseaux et al., 2006; Smith et al., 2009). Executive functions are known to be impaired in attention disorders (Nigg, 2000; Rubia and Smith, 2004; Vloet et al., 2010) and which are known to be modulated by dopamine (Konrad et al., 2004; Rubia et al., 2009).

The present study showed that 2-repeat present showed increased within-network connectivity in several regions of the ECN, including the left IFG and the left ACC. Granger causality analyses demonstrated that activity in these two brain areas mutually influenced one another (van Belle et al., 2015). They were also thought to be composed of prefrontal-subcortical circuits, which are related to task controlling and decision-making processes (Bechara et al., 2000; Power and Petersen, 2013). Furthermore, it was reported that DRD4 2-repeat allele might influence decision making performance (Ha et al., 2009), which largely depended on the prefrontal-subcortical circuits related to executive function (Brand et al., 2007). Using biotic experiments, Zhong et al. (2016) reported that the rhythmic synchronization in the PFC and its connected networks were regulated by DRD4 and controlled selective attention. A meta-analysis of 10 task fMRI datasets found that the dorsal ACC and dorsal anterior insula were the “core” of the task control system (Dosenbach et al., 2006), which might further explain the effect of DRD4 2-repeat allele on the ECN in prefrontal-subcortical circuits. In addition, a meta-analysis corroborated that three clusters in the left IFG possessed functions such as motor control, language processing, semantic and phonological processing, and working memory (Liakakis et al., 2011); and this region has been suggested to constitute a reasoning center (Chiu et al., 2004). Broca’s area, a well-known functional area related to language production, is located in the left posterior of the IFG in right-handed subjects what we included.

Our results revealed another difference in the SMN with the right MFG. Disruptions in sensory-motor circuitry were identified in relation to ADHD (McLeod et al., 2014). It was assumed that the effect of the DRD4 2-repeat on the SMN was also located primarily in the lateral PFC. A meta-analysis found that a cluster of the lateral PFC in the right hemisphere was related to fine motor control (Liakakis et al., 2011). Subsequently, some studies confirmed a predominance for sensorimotor integration functions in the right hemisphere (Aron et al., 2004). More interestingly, ADHD individuals showed that they are easily attracted to new circumstances, which is a featured of attention deficit. Indeed, the right lateral PFC has been postulated to be involved with this feature (Vandenberghe et al., 1999). Furthermore, for motor tasks with a high demand on selective attention (Kemmotsu et al., 2005) or motor tasks of great difficulty (Haslinger et al., 2002), the PFC was found to be active in bilateral cerebral hemispheres. This helps to explain the difference that decreased within-network connectivity in the left IFG of the ECN, and in the right MFG of the SMN. These observations are in accordance with the idea of the mirror neuron system mediating specialized links between perception and action (Gallese et al., 1996; Rizzolatti et al., 1996).

Combined with the behavioral items, the underlying trends of positive correlations were observed in the left IFG and in the left ACC of ADHD patients in the ECN (see Table 3). Consistent with previous studies, significant correlations between connectivity strength in the ECN and hyperactivity/impulsivity symptoms was observed, while other networks failed (Kessler et al., 2014; Francx et al., 2015; Mostert et al., 2016).The IH reflected the aspects of hyperactivity and RT, followed by the WCST-NP, reflected inattention (van Belle et al., 2015) and executive functions (Steinmetz et al., 2010), which have been widely associated with ADHD and have been able to explain the problems caused by ADHD behaviors (Goyette et al., 1978; Shaw et al., 2007, 2011; Thursina et al., 2015). In accordance with this, the RT and the WCST-NP produced activation of both these two cortical and subcortical portions of PFC circuits. Therefore, the PFC was considered to be closely related to ADHD.

The deeper study found that connectivity strength of the left IFG was directly proportional to the RT scores in the 2-repeat absent group, but not in the 2-repeat present group. Moreover, connectivity strength of the right MFG was prone to have correlation to the RT scores in the 2-repeat present group. As a higher score on the RT reflected poor performance, the higher levels of left IFG activity in 2-repeat absent group were associated with decreased performance-based levels of attention. These results suggested that 2-repeat alleles disturb the neuronal circuit of the left IFG and right MFG, which makes interference in the process of a high demand on selective attention test. It might indicate a more serious state of ADHD (RT scores: 2-repeat absent group: 2.73 ± 1.06 s; 2-repeat present group: 2.83 ± 1.09 s). It could be assumed that 2-repeat alleles in ADHD patients might be associated with pathogenesis of inattention symptom, consistent with a recent study (Kim et al., 2018). The left ACC was also included in partial correlation analyses, but it failed. As we mentioned before, genetic variation may contribute to individual differences in brain functions, even in the absence of differences in behavior. However, our results showed no statistical significance in the IH, RT and WCST-NP scores of the two groups (*p* > 0.05), which may be due to the limited number of samples, in agreement with the findings of Castellanos et al. (1998; *p* > 0.05, Sample of ADHD: 41). Moreover, Shaw et al. (2007) found a statistically significant difference (*p* < 0.05 Sample of ADHD: 104). Our results support that DRD4 2-repeat allele has an intensive effect on the PFC circles and that carriers present with dysfunction in inattention symptom. Such a link remains incomplete, however, because our analyses are based on dimensional correlations. To some extent, it has provided a potential pathophysiological basis for the role of the PFC in the diagnosis of ADHD (Dickstein et al., 2006; Fernández et al., 2009).

In this study, the absence of the DRD4 2-repeat allele increased within-network connectivity in the right PCU of the DMN. DRD4 has been implicated in the deficits of self-regulation that are controlled by the DMN (Buckner et al., 2008; Mulligan et al., 2014), which were the most commonly deactivated regions during the task fMRI experiments (Damoiseaux et al., 2006; Smith et al., 2009). Neuroimaging studies in healthy subjects have shown that inadequate DMN regulation may be a cause of attention lapses during situations that require cognitive effort. Increased activation in DMN nodes, such as PCU, preceded errors on a stop-signal task (Li et al., 2007). In line with our results, Hoekzema et al. (2014) used a* post hoc* seed-based functional connectivity analysis to suggest that individuals with ADHD presented a less stringent anticorrelation between the IFG and the most negatively associated DMN region (the right PCU). These findings could explain why the right PCU increased within-network connectivity, whereas the left IFG decreased in our results. Whereas, a recent study used ICA to reveal that there were no significant functional connectivity differences in the DMN between individuals with persistent adult ADHD and controls (Mostert et al., 2016), in accordance with the developmental delay hypothesis of ADHD (Rubia et al., 2000; Rubia, 2007).

The present study should be viewed with consideration for its several strengths and limitations.

Obvious strengths were the DRD4 2-repeat allele, medicine-naïve patient samples and extensive motion correction. Our study focused on DRD4 2-repeat allele influencing brain networks of individuals with ADHD, which has been rarely studied. As mentioned previously, 2-repeat allele is more prevalent than 7-repeat allele in Asian populations (Kitayama et al., 2014; Park et al., 2014), which deserves our further attention. Only medicine-naïve patients with ADHD were included to control for the confounding effects of previous pharmacological treatment. Previous studies have demonstrated that a psychostimulant drug used to treat ADHD, methylphenidate, normalized the effects of DRD4 in PFC pyramidal neurons during the performance of cognitive tasks (Volkow et al., 2008; Peterson et al., 2009; Tomasi et al., 2011). In contrast to previous studies, we adopted the ICA, which is a data driven blinded source separation approach that allows a hypothesis-free analysis of whole-brain fMRI data and is less prone to noise (motion, scanner artifacts; Cole et al., 2010). Moreover, we applied a new SOI-GICA, which has better robustness and accuracy than traditional algorithm (Zhang et al., 2010).

A number of limitations should be acknowledged. First, although the sample size was relatively large compared with the samples in other recent studies examining the associations between DRD4 gene and complex brain networks, there was no significant difference in the clinical symptoms. The results need to be replicated to ensure their reliability. Second, only one of the well-known polymorphisms in DRD4 gene was selected for this study. It is well-known that the effect of any single gene is likely to be modest (Durston et al., 2005). Therefore, further investigations of multiple gene linkages analyses may be necessary to more definitively determine the effect of the DRD4 gene on brain networks in ADHD. Third, only ADHD patients were recruited in the present study, which restricted further interpretation of our results. Future studies should use case-control studies to verify the role of DRD4 2-repeat allele in ADHD and to provide more valuable clinical relevance. Despite these limitations, this was the first study reporting the effect of DRD4 2-repeat allele on ADHD brain networks by means of ICA.

## Conclusion

ADHD is a syndrome that affects the entire brain networks. Overall, DRD4 gene polymorphisms, specifically 2-repeat allele, have been shown to have an effect on the ECN, the SMN and the DMN had been found. With a sample of Asian medicine-naive children with differential DRD4 genotypes, we found DRD4 mainly affect the PFC circuits, the region that controls selective attention and executive functions. In our study, ADHD children with DRD4 2-repeat allele have aberrant resting-state within-network connectivity patterns in the left IFG and the right MFG related to dysfunction in inattention symptom. This study provided novel insights into the neural mechanisms underlying the effect of DRD4 2-repeat allele on ADHD by the expression of endophenotype.

## Author Contributions

CC, CY, JL and MW designed the experiments. XW, HL, QY and LJ performed the experiments. AQ and JT analyzed the data. AQ, JC, KZ and MW wrote the article.

## Conflict of Interest Statement

The authors declare that the research was conducted in the absence of any commercial or financial relationships that could be construed as a potential conflict of interest.

## Footnotes

^1^http://www.nitrc.org/projects/cogicat/
